# Supplementary material for: Health Impacts of Ambient Biomass Smoke in Tasmania, Australia
Source: Int J Environ Res Public Health. 2020 May 7;17(9):3264. doi: 10.3390/ijerph17093264 (PMC7246513; doi:10.3390/ijerph17093264)
Supplement: Supplementary file 1 [file ijerph-17-03264-s001.pdf]

Supplementary Material for

Health impacts of ambient biomass smoke in Tasmania, Australia

Table of Contents

- **Figure S1.** Location of Base Line Air Network of EPA Tasmania (BLANkET) Air Quality Monitoring Stations (a) and Bureau of Meteorology (BOM) weather stations (b)
- **Figure S2.** Fine particulate matter **(**$PM_{2.5}$)concentration trends by time of the day and season (Summer, Transition, Winter) – base on data obtained from the Tasmania BLANkET air quality monitoring network [1]
- **Figure S3.** Tasmania Fire History (2010 – 2018). Total surface burnt (hectares and %) by fire type [2]
- **Table S1.** Population and health data – yearly averages
- **Table S2.** Health economic indicators
- **Table S3.** Estimated wood heater count per Statistical Area Level 3 (SA3)
- **Figure S4.** Average $PM_{2.5}$ concentration by Statistical Area Level 2 (SA2) and month over the study period of January 2010 and December 2019
- **Figure S5.** Average heating degree days (HDD) by SA2 level and month over the study period of January 2010 and December 2019
- **Table S4.** Summary statistics for Daily $PM_{2.5}$ ($\mu g/m^3$) per monitoring station – 01/01/2010 to 31/12/2019
- **Figure S6.** Monthly estimated landscape fire smoke (LFS) $PM_{2.5}$ by month
- **Figure S7.** Monthly estimated wood heater smoke (WHS) $PM_{2.5}$ by month
- **Table S5.** Average number of days per year (total and %) and 24h $PM_{2.5}$ summary statistics by day type and year
- **Figure S8.** Boxplot of estimated daily health costs by event type
- **Table S6.** Summary indicators for sensitivity analysis – all months
- **Table S7.** Summary indicators for sensitivity analysis – excluding months with predicted biomass smoke source

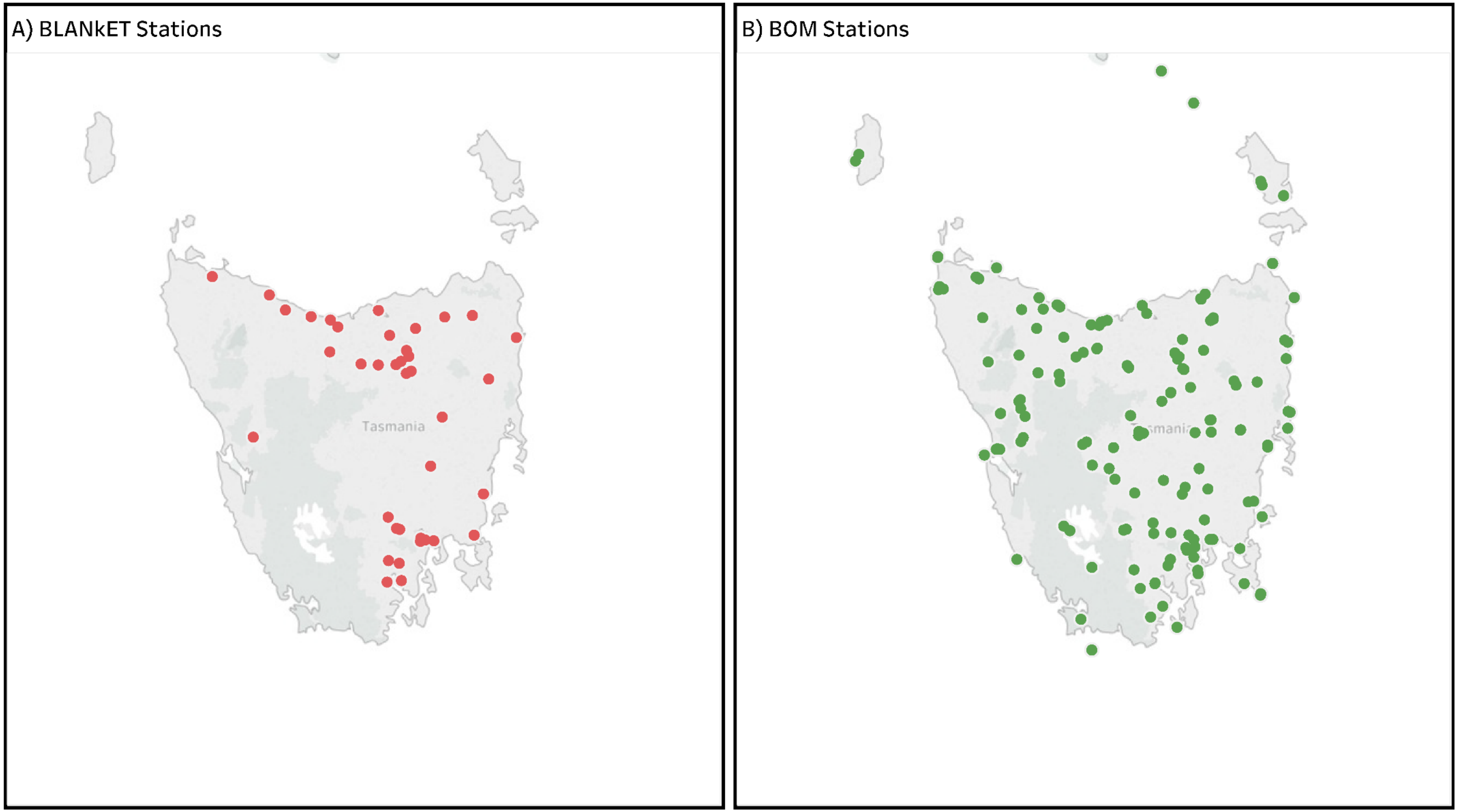

Figure S1. Location of Base Line Air Network of EPA Tasmania (BLANkET) Air Quality Monitoring Stations (a) and Bureau of Meteorology (BOM) weather stations (b)

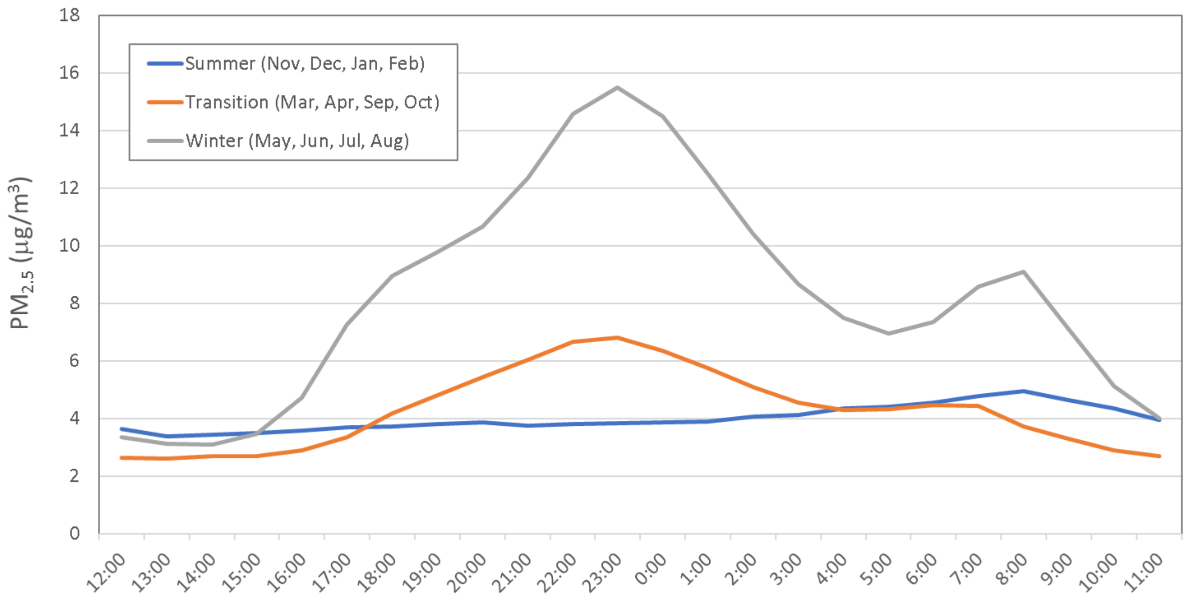

Figure S2. Fine particulate matter **(**$PM_{2.5}$)concentration trends by time of the day and season (Summer, Transition, Winter) – base on data obtained from the Tasmania BLANkET air quality monitoring network [1]. Air pollution generated by wood heaters follows a common pattern throughout the cooler months, with characteristic seasonal and diurnal patterns with a large peak overnight, and smaller peak in the early morning [3–6]

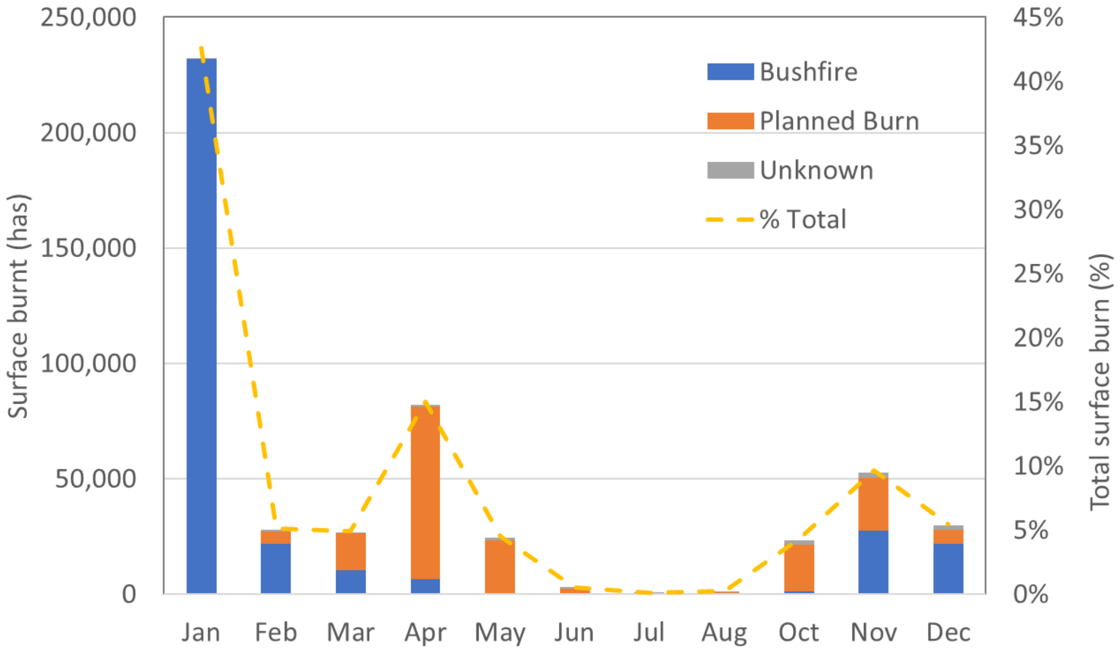

Figure S3. Tasmania Fire History (2010 – 2018). Total surface burnt (hectares and %) by fire type [2] Landscape fire activity exhibits strong seasonal patterns. Historical data demonstrate that between 2010 and 2018 bushfires dominate during January (42.5% of total hectares burnt) and planned burns during April (13.7% of total hectares burnt) (**Error! Reference source not found.**). For June, July and August there are almost no records of fires with less than 1% of total hectares being burnt during winter.

Table S1. Population and health data – yearly averages

	Females	Males	All
Population* (persons)			
0-4 years	15,044	16,013	
5-14 years	30,739	32,812	
15-24 years	31,390	33,646	
25-34 years	30,161	29,735	
35-44 years	32,512	31,040	
45-54 years	36,564	35,142	
55-64 years	35,053	34,524	
65+ years	47,620	42,214	
All	259,083	255,127	
Deaths** (cases/year)			
0-4 years	14	21	
5-14 years	15	22	
15-24 years	19	28	
25-34 years	29	53	
35-44 years	80	114	
45-54 years	7	8	
55-64 years	165	253	
65+ years	1,841	1,731	
All	2,169	2,230	
Hospitalizations – Respiratory*** (cases/100,000 persons-year)			
0-4 years	3,271.1	5,571.8	
5-14 years	1,041.8	1,031.8	
15-24 years	1,308.5	923.4	
25-34 years	1,083.9	650.3	
35-44 years	965.8	648.0	
45-54 years	1,138.2	1,158.9	
55-64 years	1,562.8	1,302.2	
65+ years	3,329.8	4,675.7	
All	1,451.3	1,540.7	
Hospitalizations – Circulatory*** (cases/100,000 persons-year)			
0-4 years	2.3	76.5	
5-14 years	71.7	341.8	
15-24 years	67.3	445.7	
25-34 years	523.0	543.0	
35-44 years	799.5	748.1	
45-54 years	929.7	2,154.4	
55-64 years	2,078.1	4,294.8	
65+ years	6,668.5	9,967.9	
All	1,480.6	2,177.6	
Emergency Department (ED) visits – Asthma (cases/year)			
2014-15			1,372
2015-16			1,447
2016-17			1,451

*average for 2010 – 2017, ** average for 2010 – 2016, ***average for July 2013 to June 2015

Table S2. Health economic indicators

Source	Indicator	Description
WHS	Total Cost ($AUD)	Total WHS costs
	Cost per day ($AUD/WHS-day)	Average daily WHS cost
	Cost per year ($AUD/year)	Average yearly WHS cost
	Cost per woodstove-year ($AUD/woodstove-year)	Average yearly WHS cost per woodstove
LFS	Total Cost ($AUD)	Total LFS cost
	Cost per day ($AUD/LFS-day)	Average daily LFS cost
	Cost per year ($AUD/year)	Average yearly LFS cost

Note. WHS = wood heater smoke. LFS = landscape fire smoke. AUD = Australian dollars.

Table S3. Estimated wood heater count per Statistical Area Level 3 (SA3). ESTIMATED BASED ON [6] and [7]

SA3	# wood heaters
Brighton	1,877
Hobart - North East	4,432
Hobart - North West	4,399
Hobart - South and West	3,269
Hobart Inner	3,478
Sorell - Dodges Ferry	2,723
Launceston	7,527
Meander Valley - West Tamar	5,531
North East	8,796
Central Highlands (Tas.)	4,910
Huon - Bruny Island	5,253
South East Coast	2,241
Burnie - Ulverstone	5,023
Devonport	6,613
West Coast	3,245
**TOTAL**	**69,317**

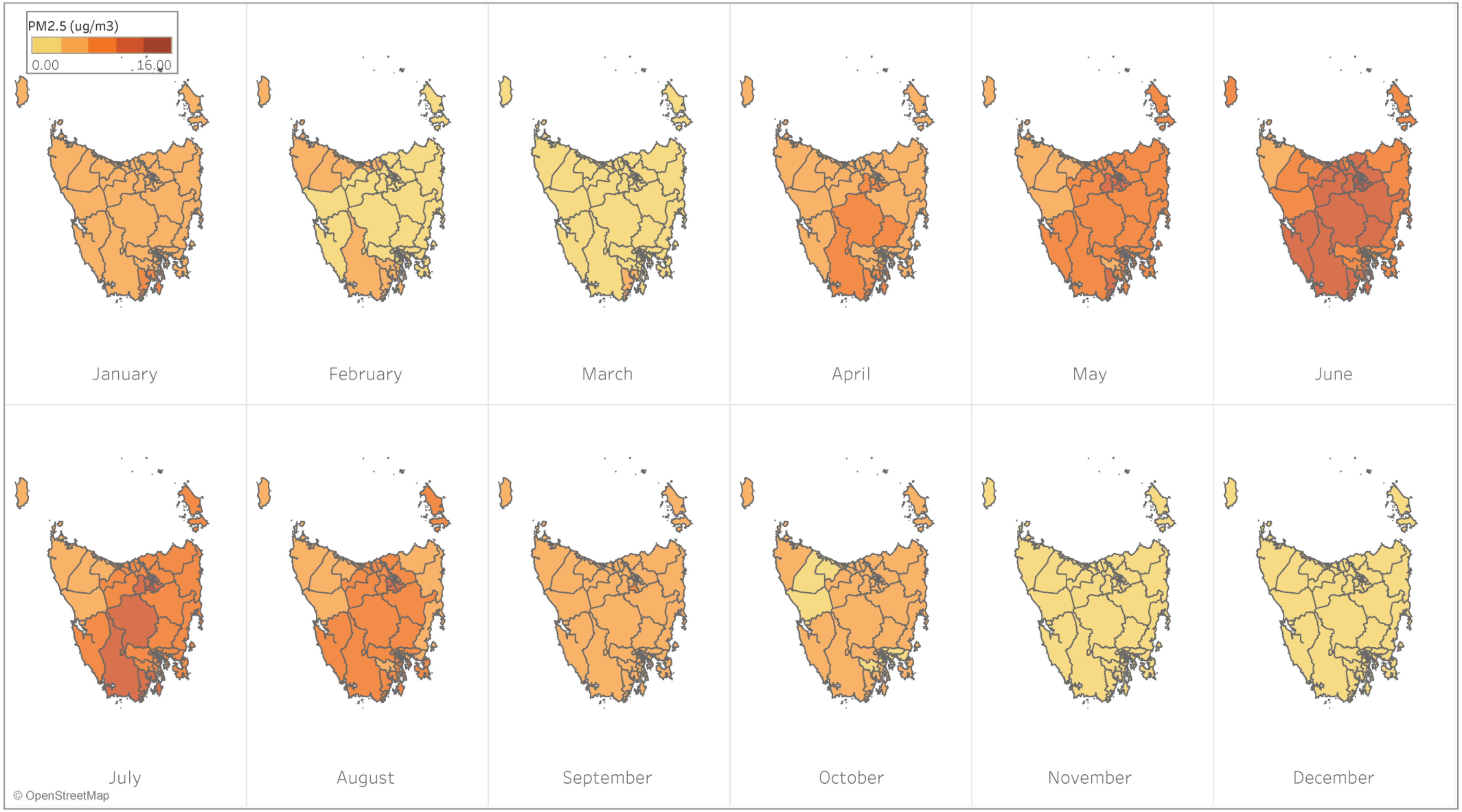

Figure S4. Average $PM_{2.5}$ concentration by Statistical Area Level 2 (SA2) and month over the study period of January 2010 and December 2019

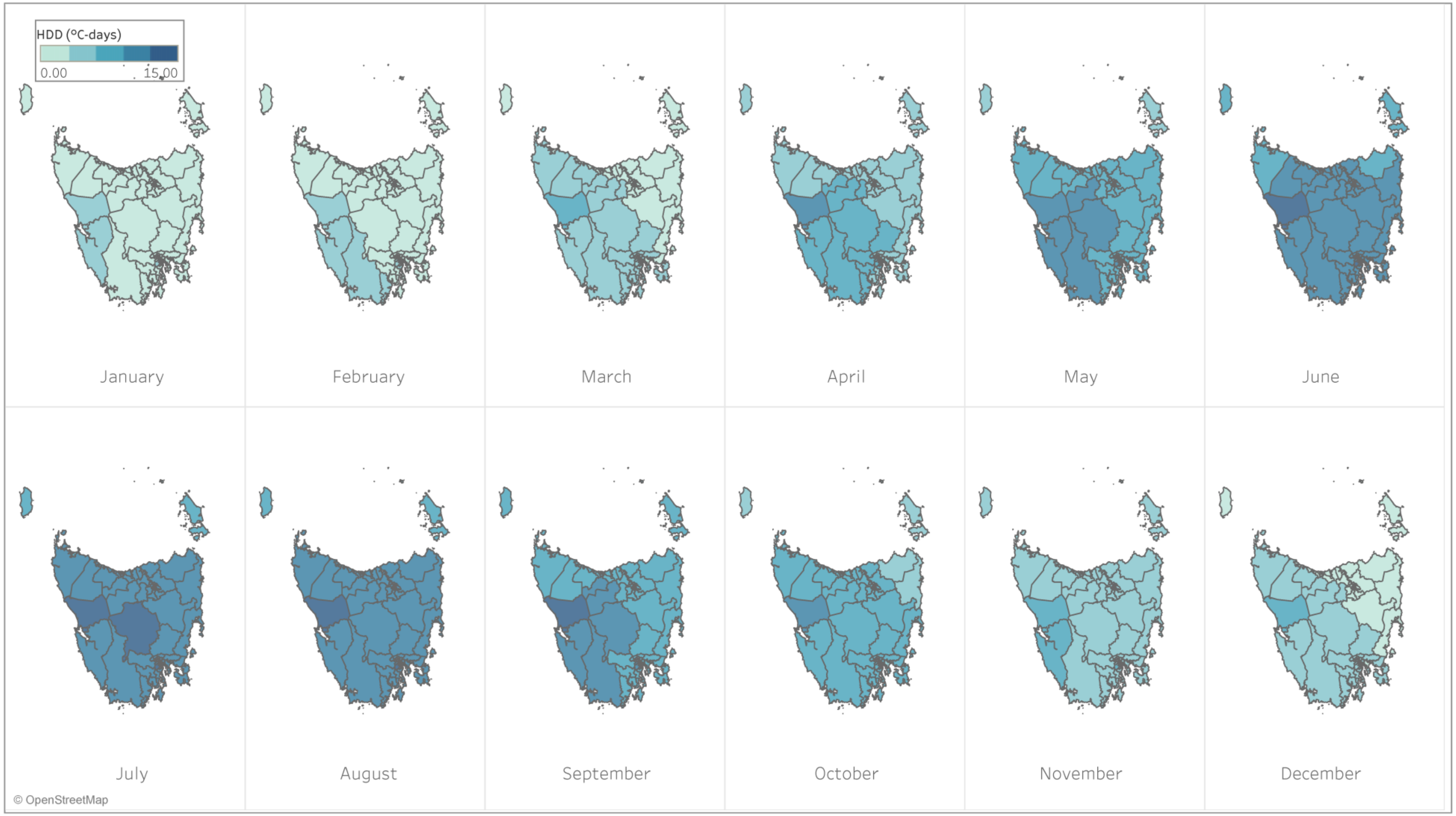

Figure S5. Average heating degree days (HDD) by SA2 level and month over the study period of January 2010 and December 2019

Table S4. Summary statistics for Daily $PM_{2.5}$ ($\mu g/m^3$) per monitoring station – 01/01/2010 to 31/12/2019

Station_name	COUNT	MIN	AVG	MAX
**Bream Creek**	1,906	0.3	2.9	42.0
**Bryn Estyn**	1090	0.4	3.4	40.5
**Carrick**	1036	0.4	3.4	29.0
**Clearys Gates**	908	0.4	3.3	31.9
**Campbell Town**	2,574	0.3	5.4	60.8
**Cygnet**	1214	0.2	4.9	350.6
**Derby**	3,518	0.2	3.6	52.7
**Deloraine**	1,738	0.3	7.4	48.6
**Devonport**	2,152	0.4	5.3	119.6
**Emu River**	3,383	0.2	2.5	206.4
**Exeter**	3,472	0.3	3.5	290.0
**Fingal**	3,472	0.2	3.1	67.0
**George Town**	2,964	0.4	4.3	145.7
**Glenorchy**	1236	0.3	4.4	55.8
**Gretna**	3,387	0.3	3.2	86.2
**Geeveston**	3,550	0.5	10.3	506.3
**Hadspen**	2,439	0.4	9.7	137.5
**Hobart**	3,486	0.0	4.6	58.5
**Huonville**	3,467	0.0	6.4	148.3
**Judbury**	3,406	0.1	2.6	194.2
**Lilydale**	3,510	0.3	4.7	94.3
**Longford**	2,404	0.5	10.9	183.6
**Latrobe**	293	1.0	9.2	67.3
**Mornington**	2,191	0.1	3.3	46.9
**New Norfolk**	2,596	0.3	9.9	67.6
**Oatlands**	1231	0.5	6.0	31.1
**Perth**	2,292	0.3	9.2	140.4
**Poatina**	1087	0.2	2.8	74.2
**Queenstown**	623	0.7	7.0	43.5
**Scottsdale**	3,541	0.2	4.3	79.3
**Sheffield**	3,431	0.1	4.0	155.5
**St Helens**	3,516	0.2	2.6	141.0
**South Launceston**	3,368	0.4	6.2	153.1
**Smithton**	3,073	0.5	4.6	316.0
**Ti Tree Bend (Launceston)**	1004	0.4	5.6	49.1
**Triabunna**	1033	0.5	6.7	129.8
**Westbury**	1051	0.6	8.1	42.8
**West Ulverstone**	1045	0.4	3.6	33.0
**Wynyard**	1046	0.6	5.7	42.1

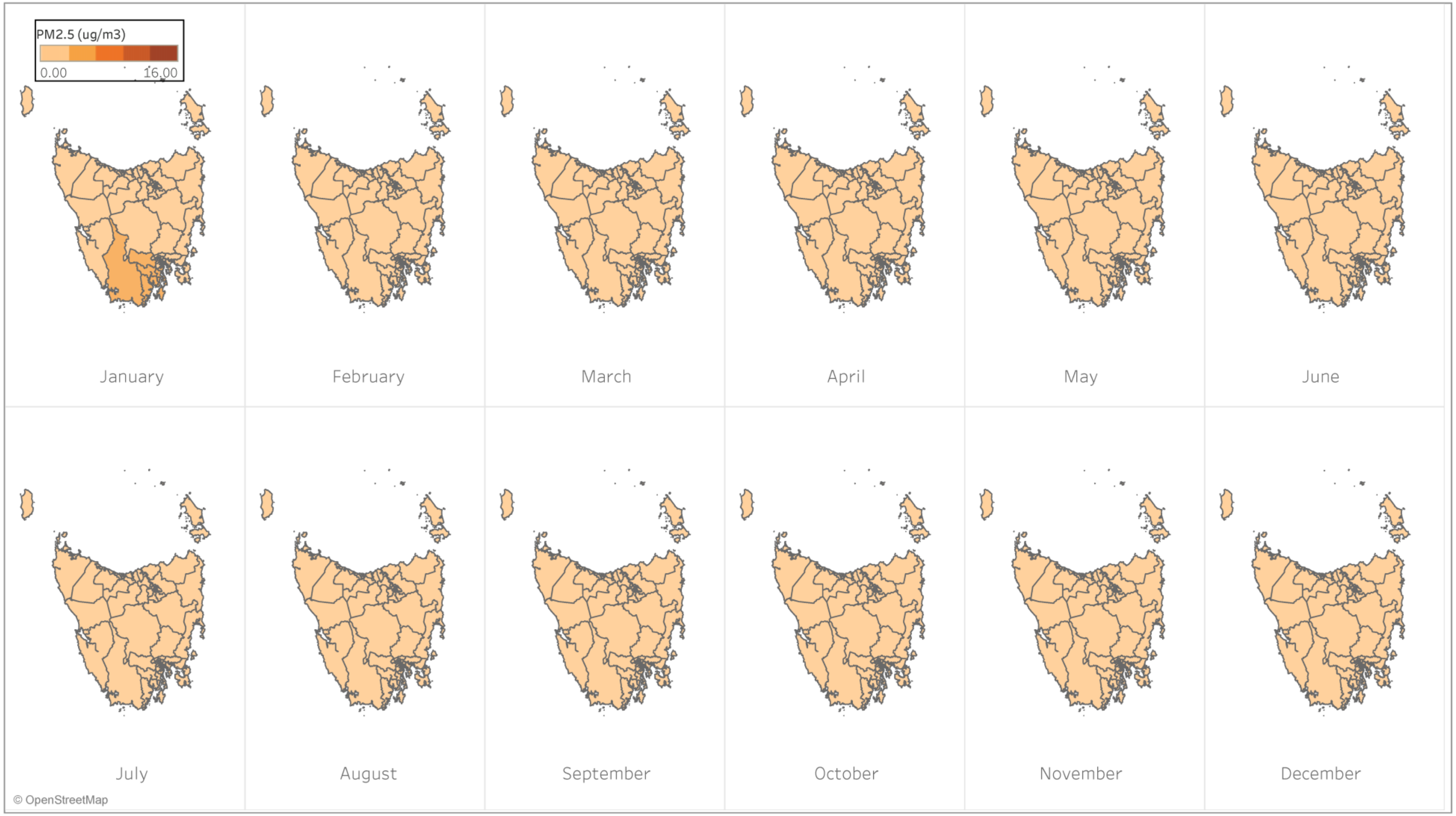

Figure S6. Monthly estimated landscape fire smoke (LFS) $PM_{2.5}$ fraction by month

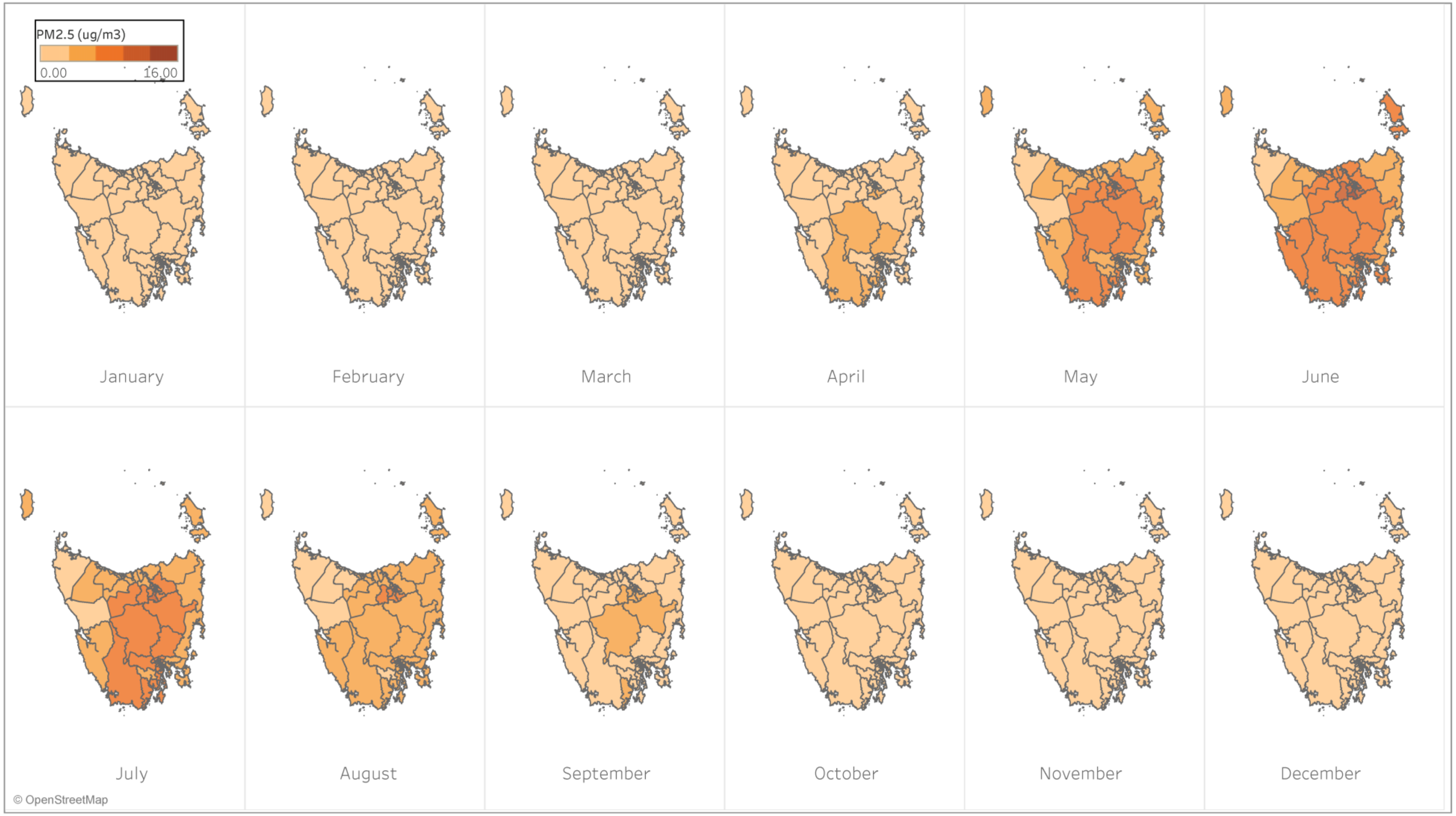

Figure S7. Monthly estimated wood heater smoke (WHS) $PM_{2.5}$ fraction by month

Table S5. Average number of days per year (total and %) and 24h $PM_{2.5}$ summary statistics by day type and year

Day type	Statistic	2010	2011	2012	2013	2014	2015	2016	2017	2018	2019
Unpolluted	Average days per year (# days)	110.3	107.9	106.2	89.4	79.9	94.2	92.1	82.5	93.2	94.6
	Average days per year (%)	30.2%	29.6%	29.0%	24.5%	21.9%	25.8%	25.2%	22.6%	25.5%	25.9%
	Mean (mg/m3)	1.6	1.5	1.4	1.4	1.6	1.4	1.5	1.2	1.3	1.3
	SD (mg/m3)	0.4	0.3	0.4	0.4	0.4	0.4	0.4	0.5	0.5	0.5
	Max (mg/m3)	3.1	2.2	2.4	2.3	2.7	2.4	2.3	2.4	2.2	2.8
LFS	Average days per year (# days)	111.9	102.6	111.8	109.5	121.5	103.3	120.0	118.4	108.4	110.5
	Average days per year (%)	30.7%	28.1%	30.5%	30.0%	33.3%	28.3%	32.8%	32.4%	29.7%	30.3%
	Mean (mg/m3)	4.1	3.1	3.5	4.2	4.1	3.6	6.3	3.6	3.1	6.3
	SD (mg/m3)	3.2	1.3	1.5	5.5	2.5	1.7	12.9	3.2	1.4	11.9
	Max (mg/m3)	52.0	14.7	20.9	142.7	33.5	32.1	312.8	38.5	39.5	237.8
WHS	Average days per year (# days)	142.8	154.4	148.0	166.0	163.6	167.5	153.9	164.2	163.4	159.5
	Average days per year (%)	39.1%	42.3%	40.4%	45.5%	44.8%	45.9%	42.0%	45.0%	44.8%	43.7%
	Mean (mg/m3)	7.7	6.0	6.5	8.7	9.4	8.2	7.1	8.4	7.8	6.8
	SD (mg/m3)	6.5	3.7	4.0	5.9	5.8	6.0	5.3	5.3	5.5	4.6
	Max (mg/m3)	168.4	33.1	40.7	46.0	47.5	54.1	109.1	51.5	81.5	48.3

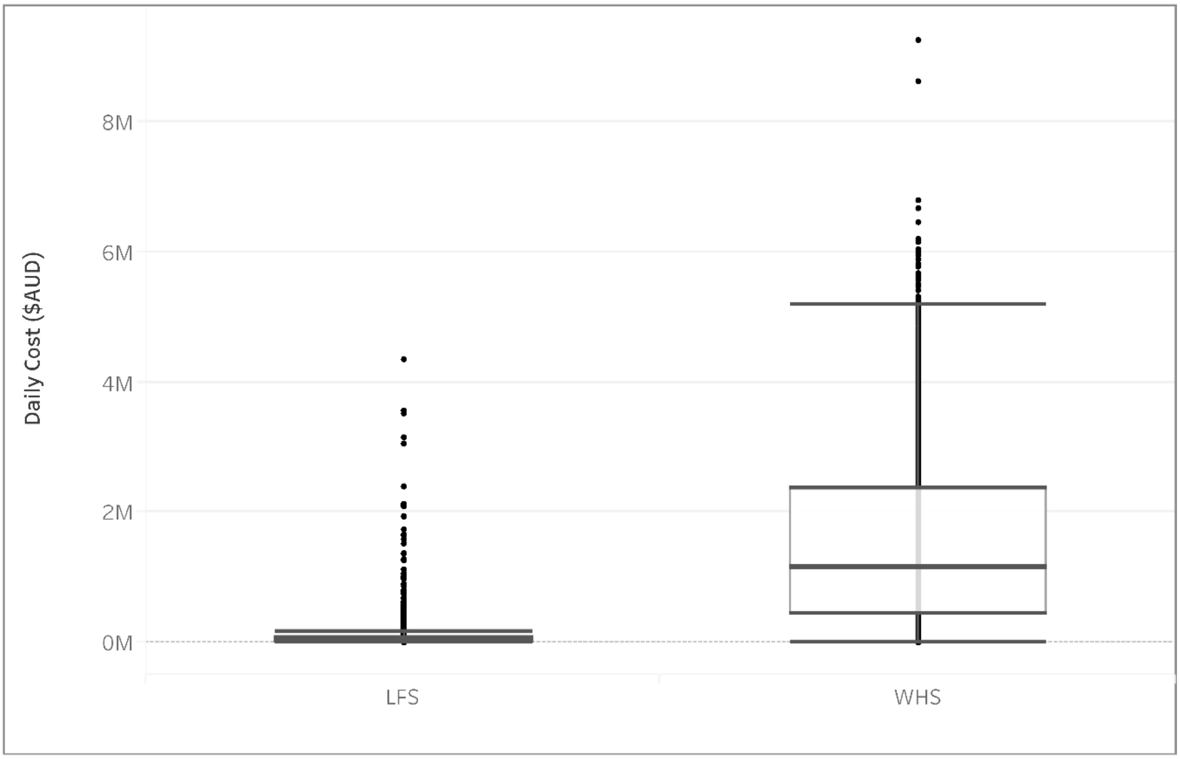

Figure S8. Boxplot of estimated daily health costs by event type

Table S6. Summary indicators for sensitivity analysis – all months

Scenario	PM2.5 threshold for LFS summer day	Start of winter	End of winter	Costs - Woodstove	Costs per day - Woodstove	Costs per year - Woodstove	Costs per woodheater-year	Costs - LFS	Costs per day - LFS	Costs per year - LFS
				$AUD	$AUD/wHS-day	$AUD/year	$AUD/woodheater-year	$AUD	$AUD/LFS-day	$AUD/year
1	P75	May	July	3,189,476,033	1,712,930	318,947,603	4,601	193,640,431	89,898	19,364,043
2	P75	May	August	3,175,192,513	1,697,964	317,519,251	4,581	196,512,399	92,870	19,651,240
3	P75	June	July	2,813,680,667	1,656,080	281,368,067	4,059	270,285,179	109,250	27,028,518
4	P75	June	August	2,905,662,596	1,677,634	290,566,260	4,192	251,361,050	106,690	25,136,105
5	P90	May	July	3,033,500,220	1,629,162	303,350,022	4,376	169,461,316	78,673	16,946,132
6	P90	May	August	3,018,845,705	1,614,356	301,884,570	4,355	172,414,756	81,481	17,241,476
7	P90	June	July	2,680,121,757	1,577,470	268,012,176	3,866	241,519,076	97,623	24,151,908
8	P90	June	August	2,765,620,955	1,596,779	276,562,096	3,990	223,926,308	95,045	22,392,631
9	P95	May	July	2,948,444,800	1,583,483	294,844,480	4,254	157,763,123	73,242	15,776,312
10 **(*)**	P95	May	August	2,933,796,608	1,568,875	293,379,661	4,232	160,717,637	75,954	16,071,764
11	P95	June	July	2,607,636,689	1,534,807	260,763,669	3,762	227,242,753	91,852	22,724,275
12	P95	June	August	2,689,558,900	1,552,863	268,955,890	3,880	210,385,117	89,298	21,038,512
13	P99	May	July	2,771,627,129	1,488,522	277,162,713	3,998	138,189,052	64,155	13,818,905
14	P99	May	August	2,757,319,383	1,474,502	275,731,938	3,978	141,076,679	66,671	14,107,668
15	P99	June	July	2,457,557,174	1,446,473	245,755,717	3,545	202,184,272	81,724	20,218,427
16	P99	June	August	2,532,237,024	1,462,031	253,223,702	3,653	186,814,521	79,293	18,681,452

**Min**	2,457,557,174	1,446,473	245,755,717	3,545	138,189,052	64,155	13,818,905
**Max**	3,189,476,033	1,712,930	318,947,603	4,601	270,285,179	109,250	27,028,518
**Main**	2,933,796,608	1,568,875	293,379,661	4,232	160,717,637	75,954	16,071,764

(*) Main analysis – all months

Table S7. Summary indicators for sensitivity analysis – excluding months with predicted biomass smoke source

Scenario	PM2.5 threshold for LFS summer day	Start of winter	End of winter	Costs - Woodstove	Costs per day - Woodstove	Costs per year - Woodstove	Costs per woodheater-year	Costs - LFS	Costs per day - LFS	Costs per year - LFS
				$AUD	$AUD/wHS-day	$AUD/year	$AUD/woodheater-year	$AUD	$AUD/LFS-day	$AUD/year
1	P75	May	July	2,095,087,377	2,277,269	209,508,738	3,022	117,298,133	97,586	11,729,813
2	P75	May	August	2,615,387,526	2,126,331	261,538,753	3,773	117,298,133	97,586	11,729,813
3	P75	June	July	1,451,117,999	2,378,882	145,111,800	2,093	117,298,133	97,586	11,729,813
4	P75	June	August	1,971,418,149	2,142,846	197,141,815	2,844	117,298,133	97,586	11,729,813
5	P90	May	July	2,008,903,175	2,183,590	200,890,318	2,898	103,746,009	86,311	10,374,601
6	P90	May	August	2,501,129,844	2,033,439	250,112,984	3,608	103,746,009	86,311	10,374,601
7	P90	June	July	1,393,520,008	2,284,459	139,352,001	2,010	103,746,009	86,311	10,374,601
8	P90	June	August	1,885,746,677	2,049,725	188,574,668	2,720	103,746,009	86,311	10,374,601
9	P95	May	July	1,961,487,669	2,132,052	196,148,767	2,830	97,269,098	80,923	9,726,910
10 **(*)**	P95	May	August	2,438,431,608	1,982,465	243,843,161	3,518	97,269,098	80,923	9,726,910
11	P95	June	July	1,361,744,516	2,232,368	136,174,452	1,965	97,269,098	80,923	9,726,910
12	P95	June	August	1,838,688,455	1,998,574	183,868,846	2,653	97,269,098	80,923	9,726,910
13	P99	May	July	1,860,035,105	2,021,777	186,003,510	2,683	85,999,700	71,547	8,599,970
14	P99	May	August	2,305,777,124	1,874,616	230,577,712	3,326	85,999,700	71,547	8,599,970
15	P99	June	July	1,293,866,903	2,121,093	129,386,690	1,867	85,999,700	71,547	8,599,970
16	P99	June	August	1,739,608,922	1,890,879	173,960,892	2,510	85,999,700	71,547	8,599,970

**Min**	1,293,866,903	1,874,616	129,386,690	1,867	85,999,700	71,547	8,599,970
**Max**	2,615,387,526	2,378,882	261,538,753	3,773	117,298,133	97,586	11,729,813
**Main**	2,438,431,608	1,982,465	243,843,161	3,518	97,269,098	80,923	9,726,910

(*) Main analysis – excluding months with predicted biomass smoke source

References

1. EPA Tasmania Base Line Air Network of EPA Tasmania (BLANkET) Available online: https://epa.tas.gov.au/epa/air/monitoring-air-pollution/real-time-air-quality-data-for-tasmania/about-blanket (accessed on Feb 1, 2020).
2. Department of Primary Industries, Parks, W. and E.-T.G. Fire History Available online: https://www.thelist.tas.gov.au/app/content/data/geo-meta-data-record?detailRecordUID=b94d4388-995d-416a-9844-a39de2798bed (accessed on Apr 1, 2019).
3. Reisen, F.; Meyer, C.P.M.; Keywood, M.D. Impact of biomass burning sources on seasonal aerosol air quality. *Atmos. Environ.* **2013**, *67*, 437–447.
4. Johnston, F.H.; Hanigan, I.C.; Henderson, S.B.; Morgan, G.G. Evaluation of interventions to reduce air pollution from biomass smoke on mortality in Launceston, Australia: retrospective analysis of daily mortality, 1994-2007. *BMJ* **2013**, *346*, e8446.
5. Innis, J. *Overview of the BLANkET smoke monitoring network: Development and operation, 2009–2015*; 2015;
6. EPA Tasmania *Spatial distribution of wood heaters and open fires in Tasmania*; 2017;
7. Australian Bureau of Statistics 2016 Census of Population and Housing: Mesh Block Counts, Australia (2074.0) Available online: https://www.abs.gov.au/ausstats/abs@.nsf/mf/2074.0 (accessed on Jan 15, 2019).
